# The broad spectrum of application of optical coherence tomography angiography to the anterior segment of the eye in inflammatory conditions: a review of the literature

**DOI:** 10.1186/s12348-019-0184-9

**Published:** 2019-09-04

**Authors:** Francesco Pichi, Philipp Roberts, Piergiorgio Neri

**Affiliations:** 1 Eye Institute, Cleveland Clinic Abu Dhabi, PO Box 112412 Abu Dhabi, UAE; 2 Cleveland Clinic Lerner College of Medicine, Case Western Reserve University, Cleveland, USA; 3 Department of Ophthalmology and Optometry, Medical University of Vienna, Vienna, Austria

**Keywords:** Optical coherence tomography angiography, Pterygium, Iris neovascularization, Anterior uveitis

## Abstract

**Background:**

With an increased number of papers on how to interpret optical coherence tomography angiography (OCTA) findings in uveitis, the aim of this review is to assess its efficacy for the quantitative monitoring of structural and functional changes in inflamed conjunctiva and iris vessels in patients with acute anterior uveitis and iris neovascularization.

**Main body:**

OCTA, currently designed as a retinal vascular imaging system, has been recently adapted for anterior segment and showed good potential for successful imaging of the conjunctiva, the cornea, and the iris. OCTA can successfully delineate corneal vessels with substantial image quality. At the same time, it can detect changes in conjunctival and limbal vascularization and thus can be applied to pseudo-inflammatory conditions such as pterygium. Anterior segment OCTA allows analysis of iris vasculature and 3-D reconstruction of the normal iris vessels. OCTA can determined iris vessel filling defects or their flow increase, when present, secondary to inflammatory conditions. In addition, OCTA gives qualitative vessel density values that can be compared pre- and post-anti-inflammatory treatment. OCTA for imaging of the iris vasculature in health and disease is highly dependent on iris pigmentation. In both OCTA and fluorescein angiography, iris pigmentation causes vasculature imaging blockage, but OCTA provides more detailed iris vasculature images. Fine, clinically invisible iris vessels can be visualized by OCTA in the very early stages as well as in the regressed stage of NVI. Additional studies including different iris pathologies are needed to determine the most optimal scanning parameters in OCTA of the anterior segment.

**Conclusions:**

This review aims to establish the current application of OCTA to anterior segment disorders of the eye, with an emphasis on exploring its use in iris vessel dilation seen in various forms of iritis, as a predictive factor for further episodes of inflammation. In addition, OCTA can depict neovascularization of the iris secondary to proliferative diabetic retinopathy.

## Background

Since its advent at the end of the last century, optical coherence tomography (OCT) has constantly evolved as far as resolution and sensitivity are concerned. The most relevant step forward of OCT has been its evolution from assessing the morphology of a tissue to studying its functional component, through optical coherence tomography angiography (OCTA) [1]. This is a technique that creates images of capillary networks by comparing the amount of light returned from static and mobile targets without the need for intravenous dye administration [2]. The past 3 years have witnessed an explosion in the application of this imaging method to vascular retinal diseases [3, 4], and only recently more and more studies are sprouting on OCTA in uveitis [5–7]. OCTA finds fertile soil in inflammatory eye diseases as vascular changes in the iris, retinal, and choroid are the core sites of the pathophysiology of eye inflammation.

The purpose of this review is to summarize the current OCTA application to the anterior segment of the eye, with emphasis on how to use it to assess iris vascular changes in anterior uveitis.

## Main text

### Techniques for anterior segment OCTA acquisition

**Fig. 1:**
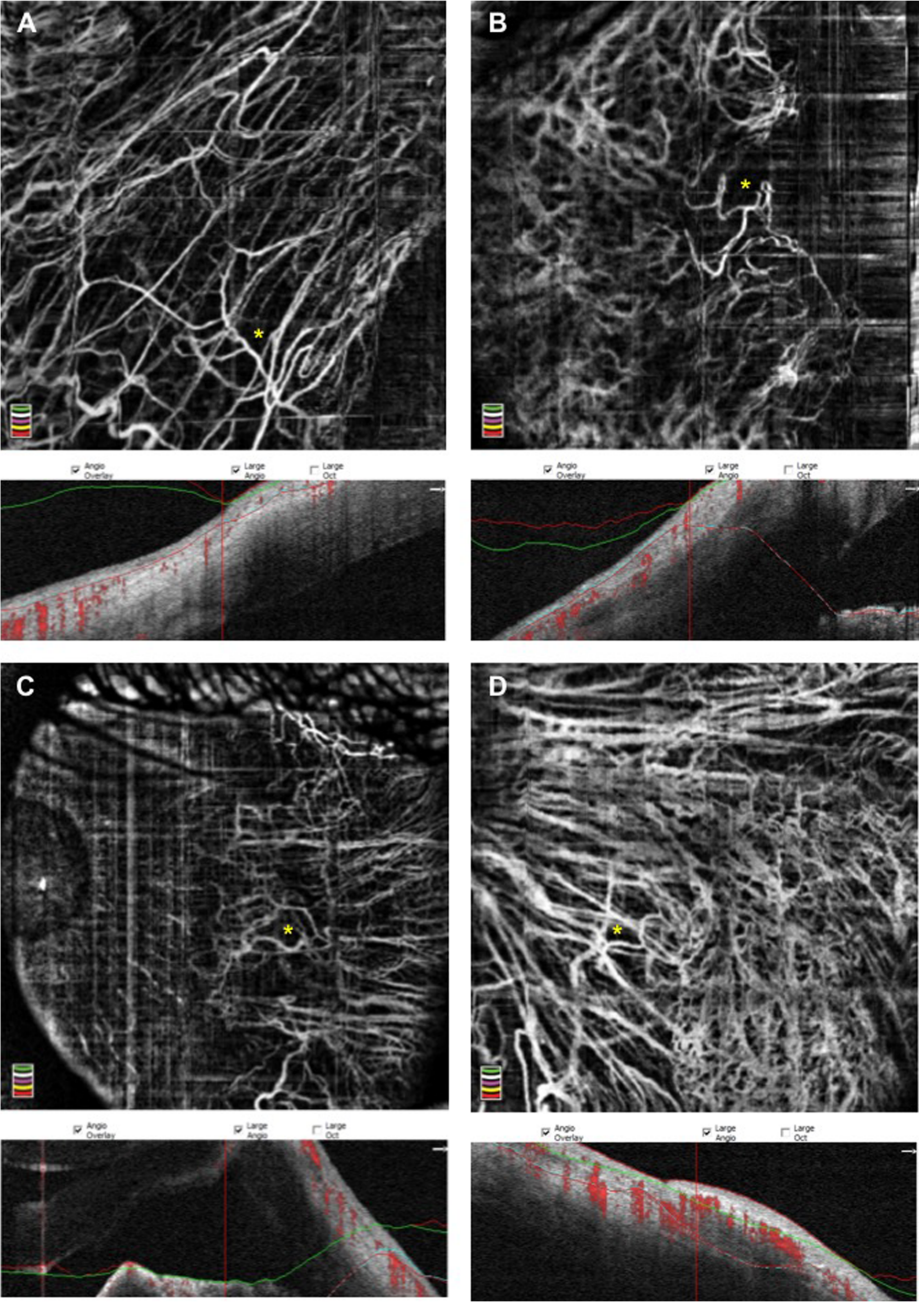
Anterior segment OCTA of four pterygia shows dilated and anomalous coiled vessels (asterisks) that differ from the smaller vessels of normal conjunctiva. Normal conjunctival vessels are less bright compared due to a slower blood flow, whereas the process of angiogenesis secondary to the para-inflammation of a pterygium increases the flow in the anomalous trunks. Scans **a**, **b**, **d** are obtained by setting the focus on + 28D, while scan **c** is obtained through an anterior segment lens. The corresponding b-scans highlight the segmentation problems secondary to the application of OCTA to the anterior segment

**Fig. 2:**
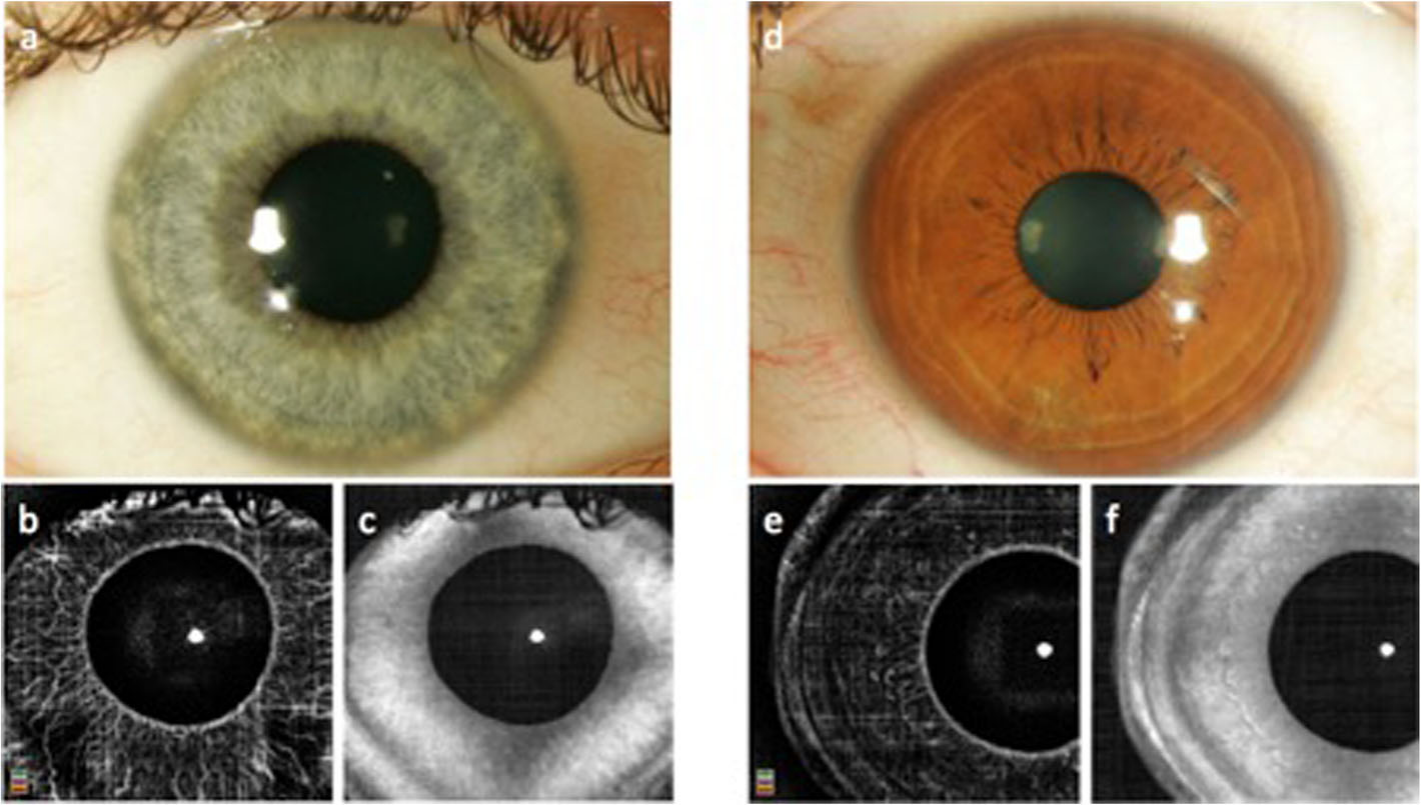
Anterior segment color photos (**a**, **d**) and en face optical coherence tomography angiography (OCTA) (**b**, **c**, **e**, **f**) of healthy eyes. Note that in the eye with blue iris color (**a**–**c**), even small vessels are visualized by OCTA (**b**). In contrast, there are no iris vessels seen in the OCTA en face image (**e**) of the eye with dark iris color (**d**–**f**). Structural en face OCT images are shown as displayed by the OCTA device (**c**, **f**)

**Fig. 3:**
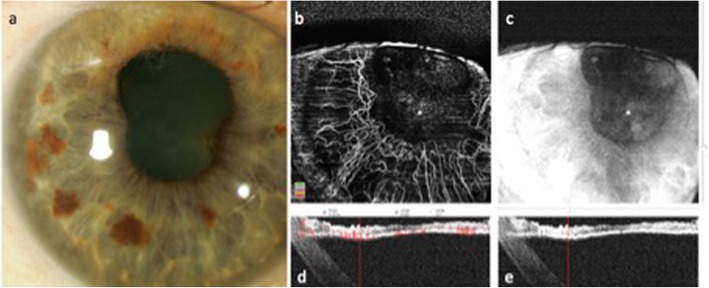
Anterior segment color photo (**a**) and optical coherence tomography angiography (OCTA) (**b**–**e**) of an eye with blue iris color and florid neovascularization of the iris (NVI). Note that B-scans (**d**, **e**) were acquired “upside down” with the iris closest to the zero-delay line (upper end of the image) to enhance image quality and to prevent imaging artifacts originating from the cornea. Despite clear visualization of iris vasculature, differentiation between healthy vessels and NVI is challenging (**b**)

For anterior segment imaging, depending on the OCTA device, an anterior segment lens may be necessary. During image acquisition, it is important to be aware of the close proximity of the anterior segment lens to the patient’s eye. To avoid artifacts originating from the cornea and to increase iris signal strength, OCTA B-scans may be flipped over the zero-delay line and be acquired “upside down” (Figs. 1c, 2b, and 3b). For some other machines, pre-dilation OCT angiography scans of the irises can be performed over a 3 × 3-mm region centered on nasal and temporal iris or over a 6 × 6-mm region encompassing the whole iris. Iris structure to be imaged can carefully positioned within the depth of focus of the scanning probe beam by manually increasing the focus settings to + 28 D.

Segmentation algorithms capable of specifically segmenting the anterior iris surface could compensate for the ambiguity in eyes with light iris color. With current technology, some effort is required to acquire and correctly interpret iris OCTA images; however, continuous improvement of imaging hardware and software will likely facilitate the use of OCTA for the anterior segment.

### Anterior segment OCTA for imaging of pterygium

Pterygium is a triangular strap-like fibro-vascular tissue that lays over the epibulbar surface of the conjunctiva, with the bottom of the triangle on the nasal conjunctiva and pointing to the cornea. Although pterygium pathophysiology is still not completely understood, it is generally accepted that ultraviolet radiations are the most important etiological factor involved in its onset, causing chronic inflammation and angiogenesis at the corneo-conjunctival junction which ultimately lead to hyperplasia accompanied by connective tissue remodeling [8].

Angiogenesis is the process of growing new blood vessels from pre-existing vessels. The vascularization process implies the activation of angiogenetic factors derived from cells. OCTA is able to detect the existence of a much richer vascularization in the pterygium than in normal conjunctiva (Fig. 1). In addition, these vessels are most evident in the subepithelial connective tissue. The morphology of these vessels is specific to neoformation: they have small caliber, are tortuous, branched, and the lumen is rarely visible. In the remaining connective tissue, the blood vessels have a regular diameter and morphology, very similar with normal conjunctive; their lumen is visible and sometimes distended, filled with blood.

### Anterior segment OCTA for imaging of the iris vasculature and iris neovascularization

Neovascularization of the iris (NVI) is a severe, sight-threatening complication of different diseases such as diabetic retinopathy (DR), retinal vein occlusion (RVO), ocular ischemic syndrome, or uveitis [9–11]. Typically, NVI are the result of either a chronic or acute ischemic insult to the retinal tissue, causing the release of pro-angiogenic factors such as VEGF which lead to neovascularization [10]. Growth of new vessels starts around the pupillary border and at the iris root. In later stages, the new vessels may cover the entire anterior iris surface, causing a red iris flush, which is also referred to “rubeosis iridis” (Fig. 2) [11]. As a consequence of NVI, rubeotic glaucoma may develop, necessitating prompt and aggressive rescue treatment. However, despite optimal treatment, successful disease management is difficult and visual prognosis is guarded in most cases [10].

Gartner and Henkind classified NVI into three stages, based on clinical presentation. In stage 1, fine and thin-walled irregular vessels may be observed at the pupillary border and at the iris root. In stage 2, these vessels enlarge and new vessels also develop in the iris stroma. In stage 3, vessel complexes merge and lead to formation of peripheral anterior synechiae, which can block aqueous outflow and thus cause ocular hypertension and glaucoma [11].

Diagnostic procedures for the diagnosis and staging of NVI include slit lamp biomicroscopy, gonioscopy, and, for the detection of subclinical stages, may also include anterior segment fluorescein angiography (FA) or indocyanine green angiography (ICGA) [11–13].

Slit lamp biomicroscopy and gonioscopy, while performed fast and easily during routine examination, requires some experience and does not offer objective documentation as a reference for follow-up examinations. Furthermore, very fine NVI may be missed. Iris FA can be helpful in cases with suspected NVI, as it shows leakage from new blood vessels [14, 15]. ICGA, in contrast, depicts vessel anatomy in more detail, since ICG molecules are mostly protein-bound and mostly remain within blood vessels. Furthermore, ICGA is performed using near-infrared imaging, which allows better penetration in pigment [13].

Both FA and ICGA, however, have the disadvantage of being invasive and time-consuming procedures associated with a number of risks such as nausea or allergic reactions.

With OCTA as a new and non-invasive diagnostic instrument for objective and high-resolution imaging of healthy iris vasculature as well as NVI could now be added to our armamentarium of imaging modalities [16].

Shortly after OCTA has been successfully applied to the cornea, it has been shown that it may also be valuable for fast and reproducible imaging of the iris vasculature.

Similar to clinical examination, it may be difficult to reliably detect and differentiate healthy iris vasculature from NVI in all cases. While in a darkly pigmented iris the normal vasculature can hardly be detected due to blockage of the probing light beam, even small iris vessels may easily be rendered in a lightly pigmented iris (Fig. 2).

**Fig. 4:**
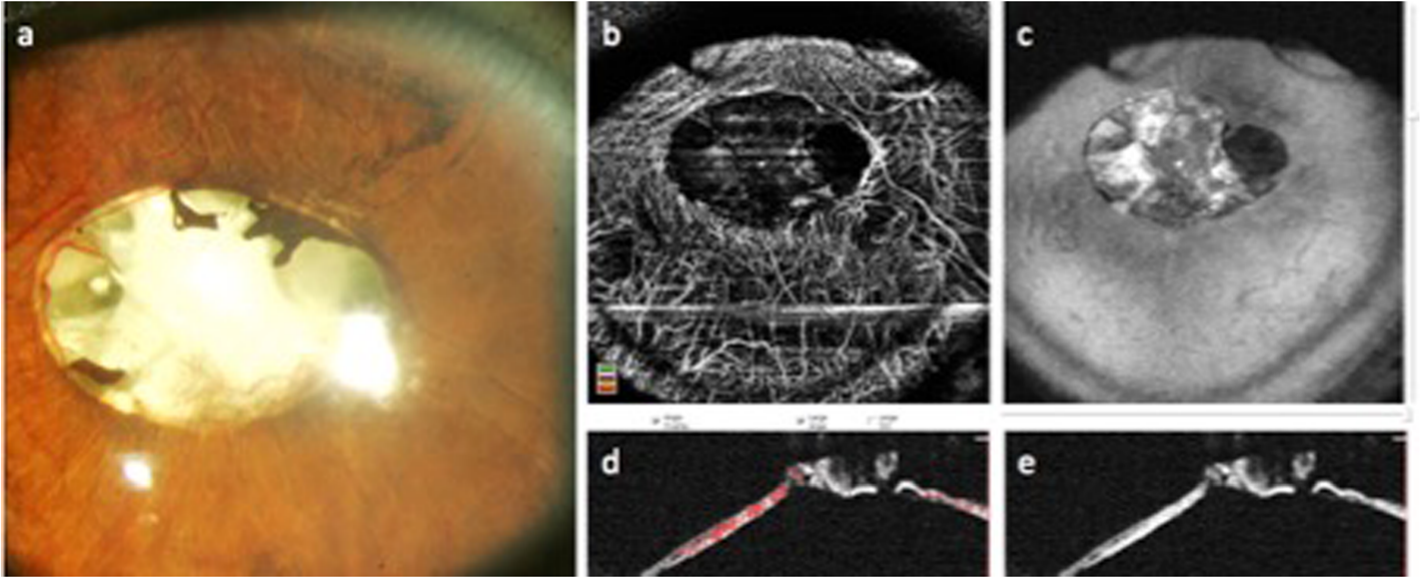
Anterior segment color photo (**a**) and optical coherence tomography angiography (OCTA) (**b**–**e**) of an eye with brown iris color and florid neovascularization of the iris (NVI). NVI can clearly be identified since healthy iris vessels are located behind the pigmented anterior border layer and are not visualized in en face OCTA (**b**)

Hence, knowledge of iris anatomy including iris vasculature is a prerequisite for a correct interpretation of en face iris OCTA images. Normal iris vessels are located in the iris stroma, covered by the anterior border layer and may not be visible in slit lamp examination in darkly pigmented eyes [17]. In contrast, NVI is located on the iris surface. On the other hand, the differentiation between healthy iris vessels and NVI may be challenging in eyes with a lightly pigmented iris, because of the overlap of physiological and pathological vessels in en face view (Fig. 3). In darkly pigmented irides, however, where normal iris vasculature is located behind the iris pigment, NVI can be diagnosed more easily (Fig. 4). This is also the case in en face OCTA imaging.

Anterior segment OCTA could, thus, not only be used for screening purposes but also as a follow-up tool to assess microvascular structural changes in response to treatment such as laser therapy or intravitreal anti-VEGF injections.

### Anterior segment OCTA for imaging of the iris vasculature in anterior uveitis

Uveitis refers to inflammation of the uveal tract of the eye [1], including the iris, ciliary body, and choroid [18]. Grading systems are currently used to quantify inflammation of the anterior and posterior chamber [19]. Anterior chamber cells, vitreous cells, vitreous haze, and scleral inflammation are graded using an ordinal-based system, utilizing clinical exam. There are several reports on utilizing OCT imaging to objectively identify and quantify anterior chamber cell [20–22] and vitreous haze.

The iris and ciliary body are the primary sites of inflammation in anterior uveitis. While the ciliary body is not visible, the iris can be easily visualized [23]. During active inflammatory episode, the iris changes, with iris vessel dilation visible. Attempts to identify and measure iris vessel dilation have been limited to anterior segment fluorescein angiography, which presents several limitations as previously mentioned [14]. However, the capillaries in the iris are in fact non-fenestrated; for this reason, fluorescein angiography studies of the iris in uveitis have not shown leakage [14, 24]. OCTA presents a possible objective measurement of ocular inflammation.

Choi et al. [25] were the first to apply OCT-based microangiography to an acute anterior uveitis model in rats. They initiated anterior uveitis in two female Lewis with an intravitreal injection of 10 μg of killed mycobacterium tuberculosis antigen. They then imaged blood flow in the irises of these rodents through an OCT angiography algorithm. Their OCT angiographic results showed that the iris vessels became dilated 2 days after induction of anterior uveitis while the limbal circulation was not affected. To quantify their amount of vascular dilation, Choi et al. [25] compared manually measured iris vessel diameters at the same location for the pre- and post-treatment OCT angiograms. On average, the vessel diameters at day 2 were 30 to 40% greater than those at day 0.

From a rodent model with manual measurements, Pichi et al. [26, 27] were the first to objectively quantify iris vasculature through OCT angiography in 35 patients with acute anterior uveitis (AAU) (idiopathic in 11 patients, HLA-B27 associated in seven patients, rheumatoid arthritis in three patients, and sarcoidosis associated in nine patients) and 40 control eyes of 20 patients. The authors performed pre-dilation OCT angiography scans of the irises with Optovue RTVue XR Avanti (Optuvue, Inc, Fremont, CA). Each subject underwent one imaging session at baseline, at day 7, and day 14, that included two sets of scans over a 3 × 3-mm region centered on nasal and temporal iris. Iris structure to be imaged was carefully positioned within the depth of focus of the scanning probe beam by manually increasing the focus settings to + 28 D.

#### Qualitative analysis

**Fig. 5:**
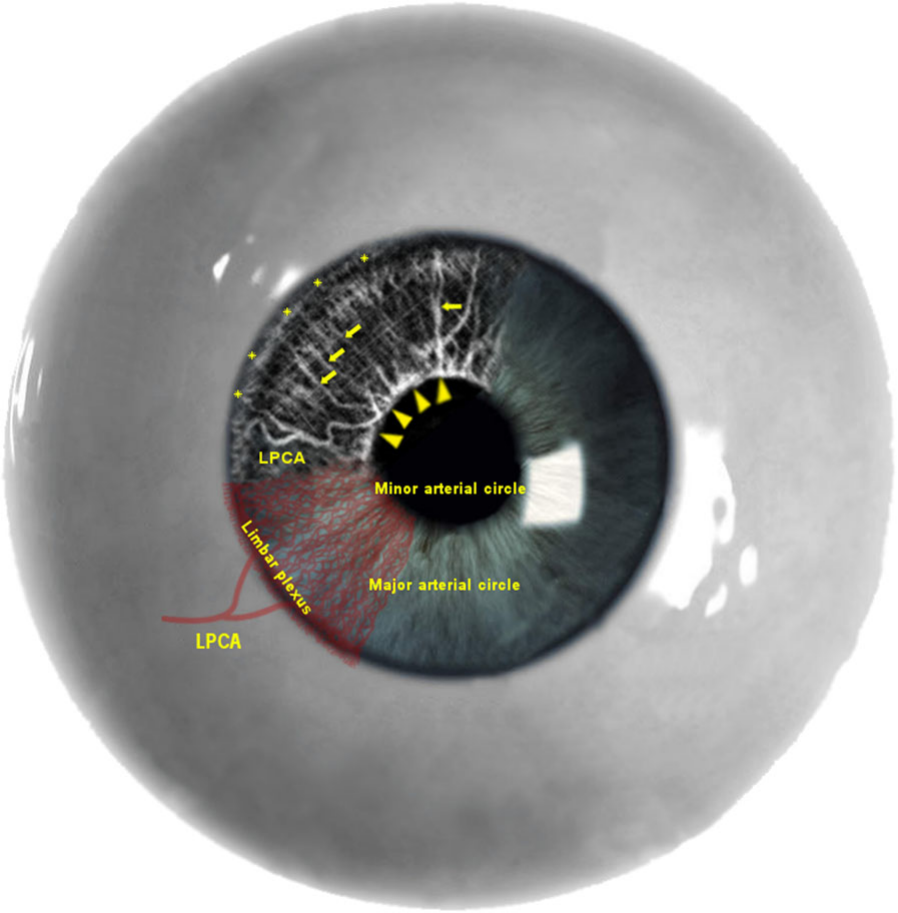
Optical coherence tomography angiography of inflamed iris highlights the anatomy of the vessels. A single branch of a long posterior ciliary artery (LPCA) in the vicinity of the iris root gives rise to a major arterial circle (arrows) traveling almost perpendicular in the iris body. In the pupillary margin, the lesser iris circles (arrow heads) are clearly visible, bridging adjacent vascular networks. At the root of the iris, a hyperreflective band (asterisks) runs almost continuously, representing the limbal circulation. Anterior to that the ciliary circulation is obscured due to the fact that the vessels dive deep towards the ciliary bodies thus being below the focal plane of the optical coherence tomography

Figure 5 shows the corresponding vascular network within the iris tissue of inflamed eyes. The iris microvasculature is clearly highlighted, with densely packed radial small vessels toward the center of pupil and irregular less densely packed vessels toward the iris root. A single branch of a long posterior ciliary artery (LPCA in Fig. 2) traveling almost perpendicular to the radial vessels could be seen in 77% of the uveitis irises in the vicinity of the iris root. Major arterial circles are observed around the iris root and the ciliary body from which small arteries (dotted arrows) branch out and extend toward the pupillary margin. In the pupillary margin, the lesser iris circles with corn-shaped arcades (arrow heads) are clearly visible, bridging adjacent vascular networks. At the root of the iris, a hyperreflective band (Fig. 5, asterisks) runs almost continuously, representing the limbal circulation. Anterior to that the ciliary circulation is obscured due to the fact that the vessels dive deep toward the ciliary bodies thus being below the focal plane in our imaging study. This baseline OCT angiogram shows that the iris vessels become dilated with anterior uveitis, and therefore they can be better highlighted.

#### Quantitative analysis

**Fig. 6:**
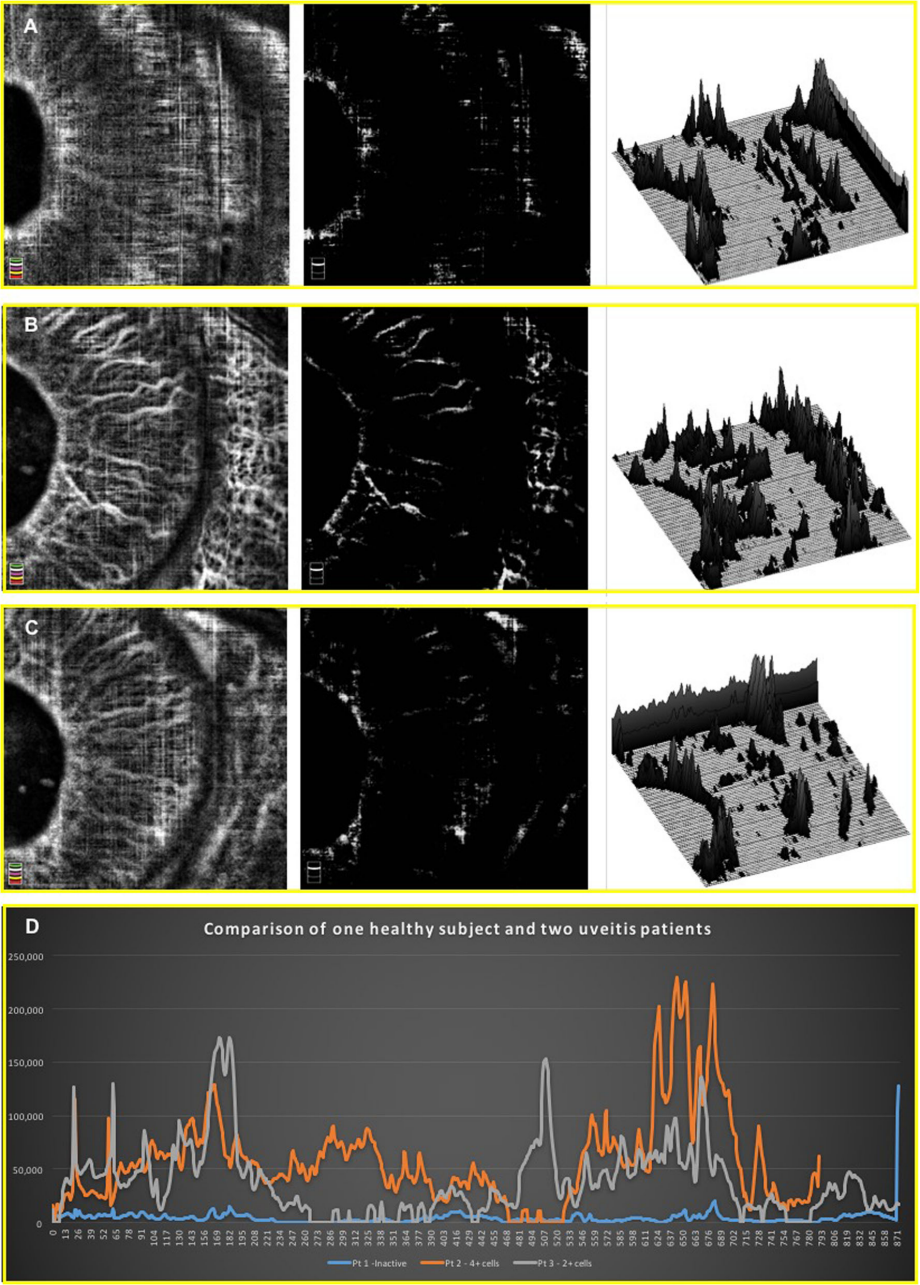
All OCTA images (left column) were converted to an 8-bit grayscale (center column) and white vessels reflectivity value was calculated in pixels. Pixels measurements of vessels reflectivity were both compared through a 3D rendering (right column), in which peaks height correspond to vessels intensity, and through a histogram (bottom panel). Iris vessels caliper and reflectivity on optical coherence tomography angiography is higher in the eye with 4+ cells (top row, orange line in the histogram), decreases in the eye with 2+ cells (second row, grey line in the histogram), and is barely detectable in a normal eye (third row, blue line in the histogram). In healthy subjects, iris vessels are scarcely visible through optical coherence tomography angiography, since the flow inside them is below the scan threshold

**Fig. 7:**
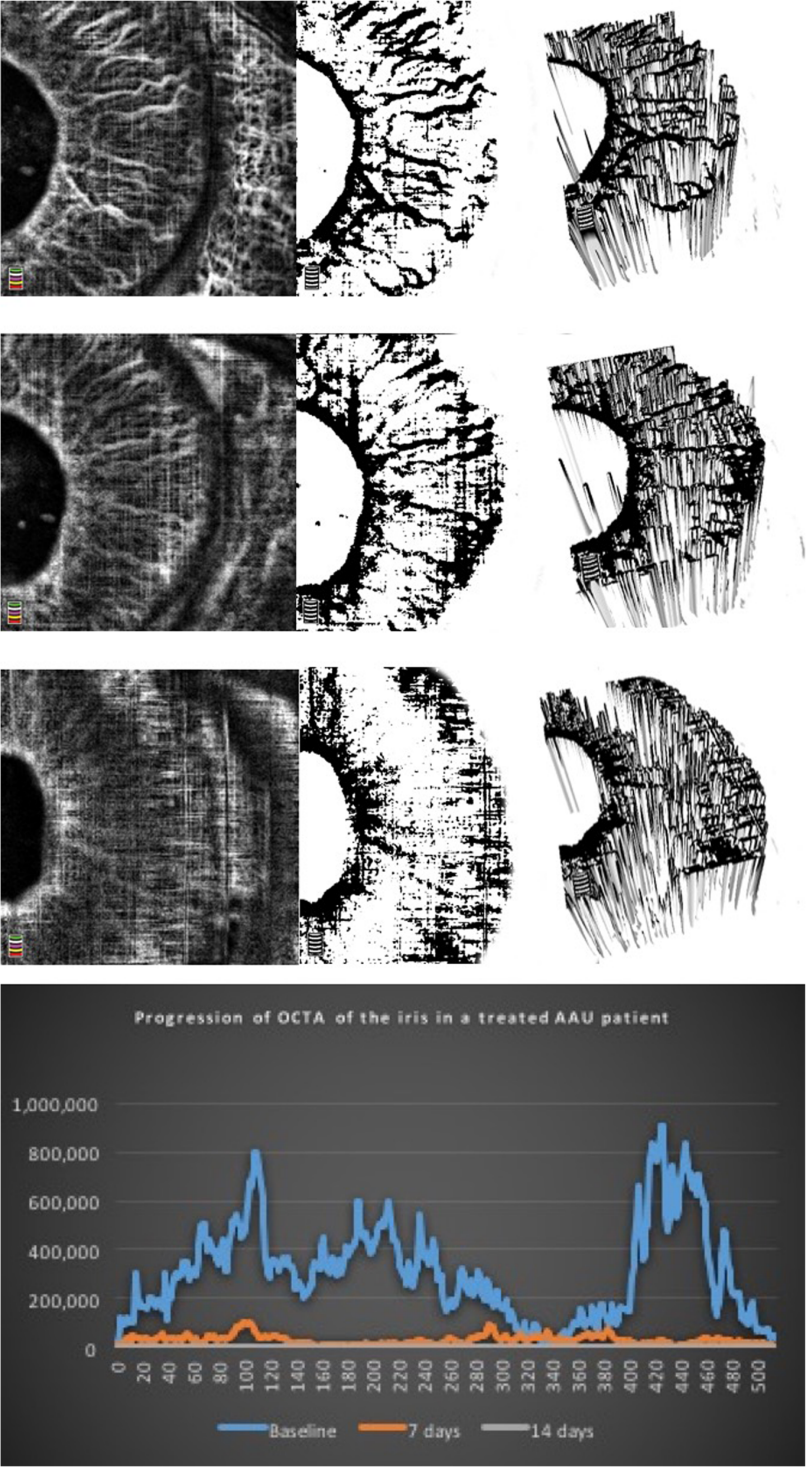
Optical coherence tomography angiography of the iris in an active anterior uveitis patient highlights progressive decrease of the vessels caliper and dilation with control of inflammation. A 36-year-old patient with acute anterior uveitis examined with optical coherence tomography angiography of the iris at baseline (top row, 4+ cells), after 7 days of prednisolone acetate 1% every 2 h (middle row, 2+ cells) and at 15 days (bottom row, quiet). The progressive in the caliper of the vessels is qualitatively visible in the greyscale rendering (middle column) and in the 3D extrusion of the reflectivity (right column), and is confirmed by the histogram (bottom) that compares point-by-point pixels reflectance of the iris vessels when inflamed (blue line), when less inflamed (orange) and quiet (flat grey line). This anterior uveitis patient is the perfect example of how optical coherence tomography angiography of the iris can detect inflammation through dilation of the iris vessels

In addition to describing the iris vessels in anterior uveitis patients, Pichi et al. [27] also investigated blood perfusion changes in the iris during acute inflammation and treatment using OCT angiography. To quantify the amount of flow increase and vessels dilation that occurs with anterior inflammation, the authors compared brightness of the grayscale renderings of pre- and post-treatment OCT angiograms. Post-image acquisition analysis on all OCTA of the iris was performed through ImageJ (https://imagej.nih.gov/ij/). The measurement procedure included obtaining the ROI within the pupil and the iris root. All images were converted to an 8-Bit grayscale calculated by converting each RGB image into a gray scale value using the following formula: *V* = 0.299R + 0.587G + 0.114B (*V* = *Y*; *R* = red; *G* = green; *B* = blue) (Hartig 2013; Kang and Kim 2014). Brightness was then adjusted in a standardized way in order to highlight only the bright vessels. Reflectivity (highlighted in white) was measured with the ImageJ software, which converts gray scale images to intensity per pixel to calculate reflectivity values (Fig. 6 and Fig. 7).

Mean brightness in heathy irises in pixels was 4.9 ± 3.5. In the uveitis group, mean brightness in eyes with 4+ cells at baseline was 61.5 ± 13.4, in eyes with 3+ cells it was 44.1 ± 9.7, in eyes with 2+ cells it was 21.8 ± 16.3, and finally in 1+cells eyes the mean brightness in pixels was 17.9 ± 7.2 (Fig. 9). Figure 6 third row presents an anterior uveitis patient with a 3+ anterior chamber inflammation at baseline; the iris vessels can be better highlighted in OCTA and the surface plot shows peaks of brightness in the greyscale rendering. Figure 7 second row shows a 2+ cells patient and the difference in brightness peaks compared to Fig. 7 first row (1+ cells). All brightness data extrapolated from the greyscale image can be graphically compared (Fig. 7 bottom row).

#### OCTA changes with treatment

Twenty patients (57.1%) had an improvement in anterior uveitis after 7 days of treatment, and the topical treatment was tapered accordingly. Fifteen patients (42.8%) did not show an improvement at day 7 so treatment was not tapered, by day 15 88.6% of patients were quiet and 11.4% had improved to 1+ cell. By day 15, there were no patients with greater than 1+ cell in the anterior chamber (Fig. 9).

**Fig. 8:**
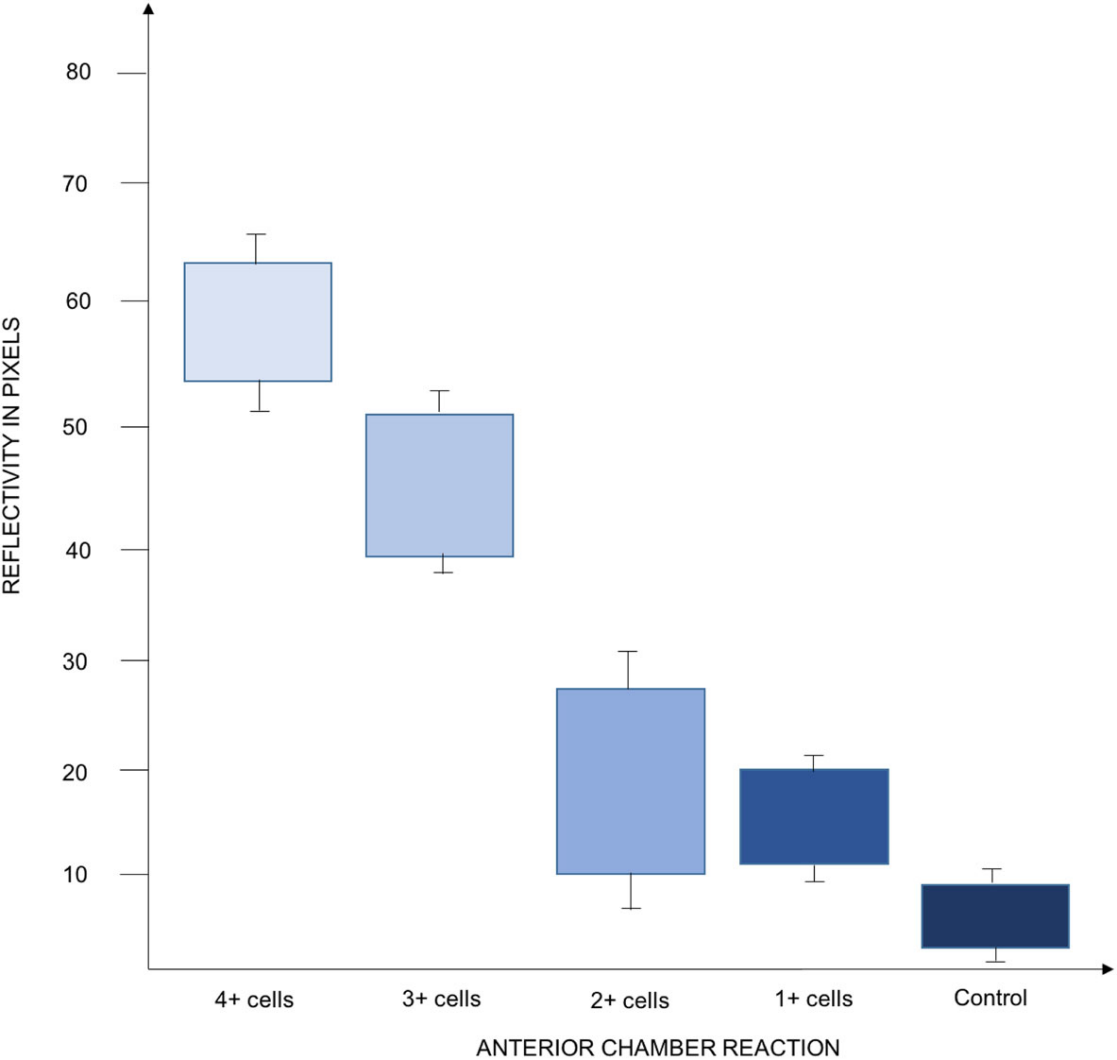
Automated algorithm to measure iris vessels volume in patients with acute anterior uveitis. A 3 × 3 OCT angiogram of the nasal iris of a patient with 3+ anterior chamber cells is shown in panel **a**. A 3D cube is processed using a combination of 3D Gaussian filters, spectral bandpass filters, intensity thresholding, and 3D morphological “opening” filters to remove non-specific, non-contiguous voxels (**b**). Connected voxels represent vessel segments. Vascular volumes were subsequently calculated by summing voxels in the resulting vessel masks (**c**, **d**)

**Fig. 9:**
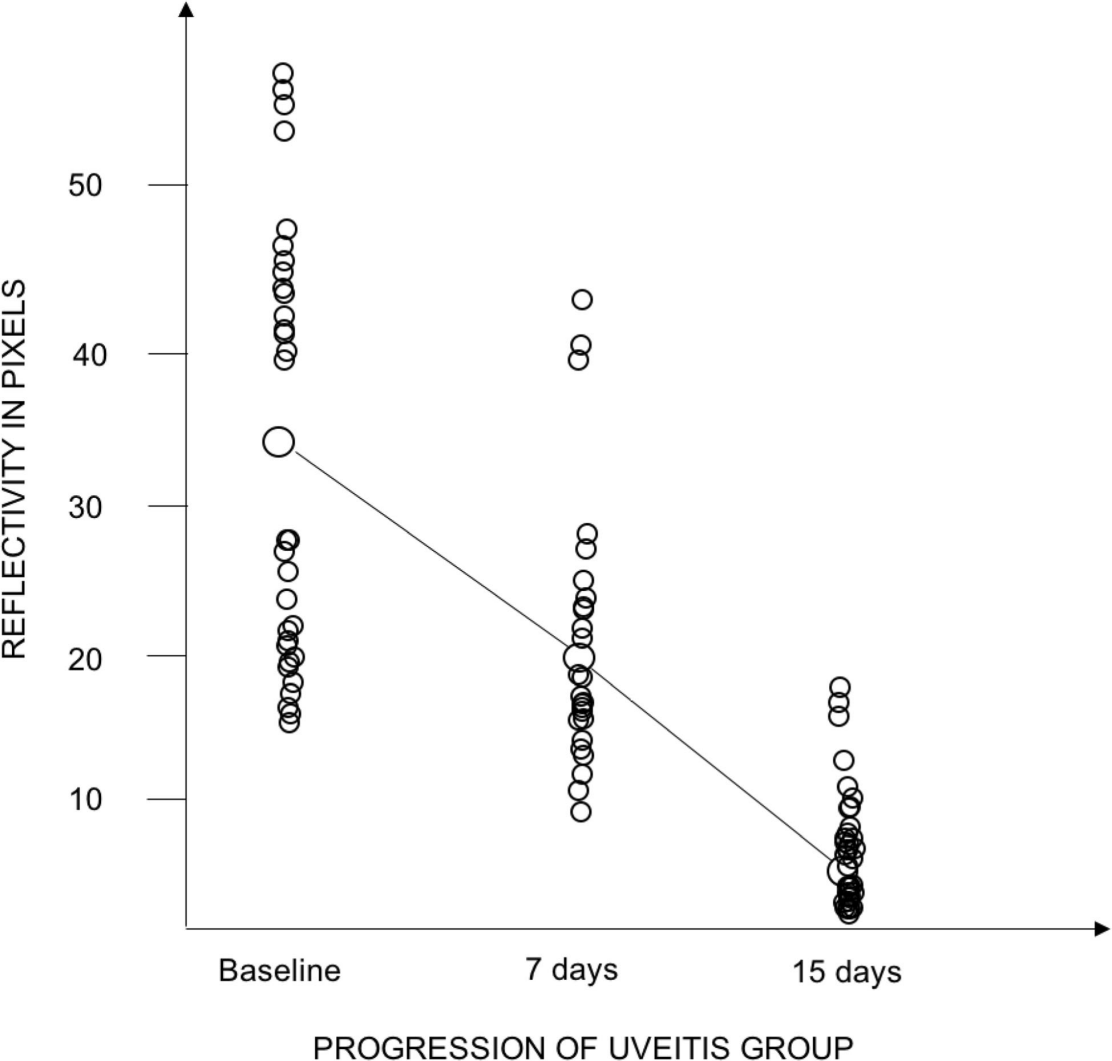
Baseline reflectivity of the iris vessels on optical coherence tomography angiography. Optical coherence tomography angiography images of the irises of the acute anterior uveitis patients and the controls were converted in 8-bit grayscale in order to analyze the vessels reflectance in pixels. The baseline pixels reflectance is reported in Fig. 8 and 9, with patients with higher inflammatory activity showing a higher vessels reflectance, and controls with a barely detectable pixels reflectance

Comparison of the iris vessels brightness measured with OCTA in the uveitis group at baseline is reported in Fig. 8 and at different follow up in Fig. 9.

**Fig. 10:**
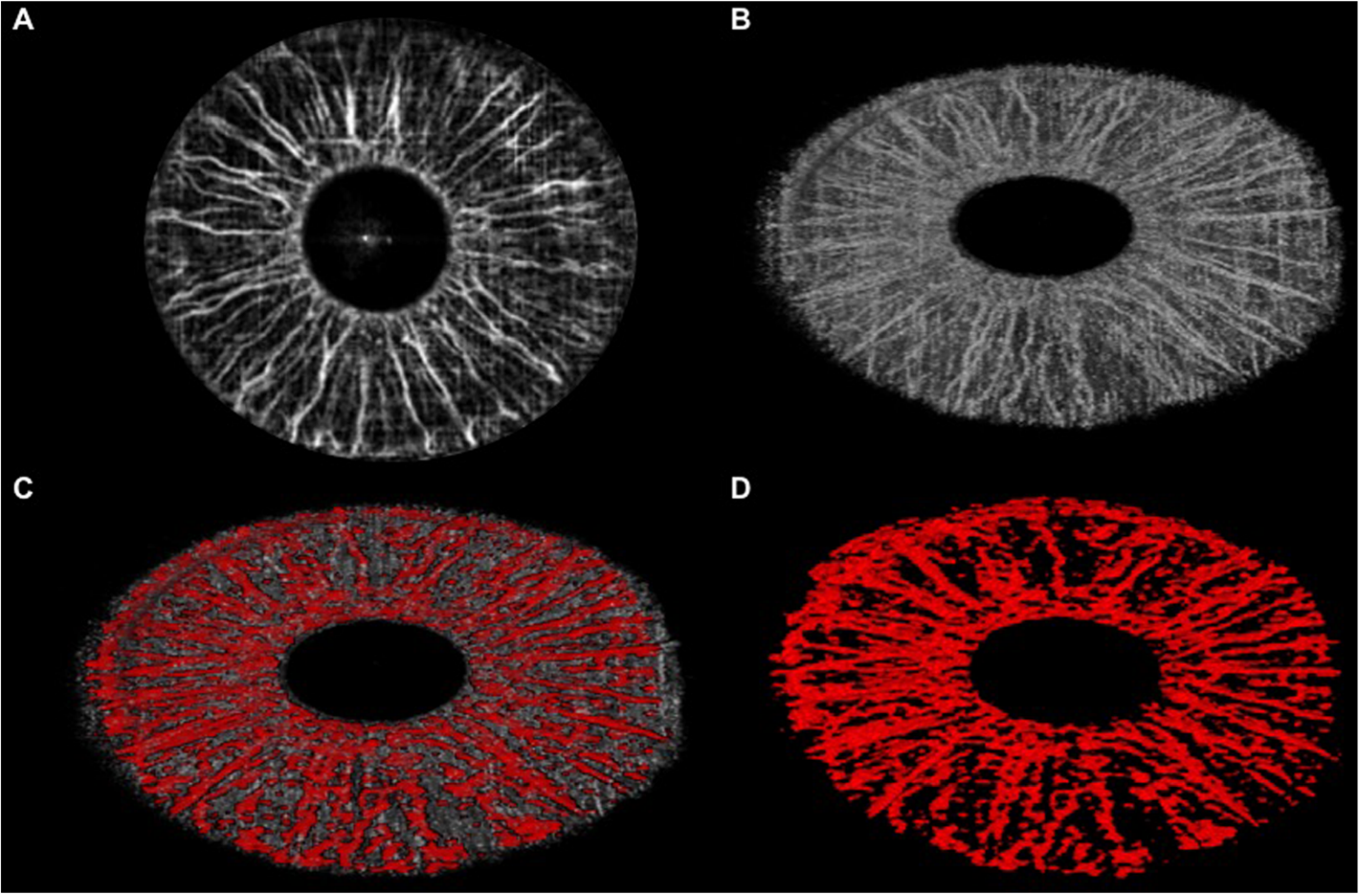
Progression of iris vessels reflectance on optical coherence tomography angiography with topical treatment in acute anterior uveitis patients. Iris vessels reflectance on optical coherence tomography angiography in 35 uveitis patients decreases from baseline, to 7 and 15 days post-treatment, together with inflammation control

Figure 7 highlights this change in an anterior uveitis patient with 4+ baseline cells (top row), that improved to 2+ cells at 7 days (second row) and was completely quiet at 14 days (third row). The progressive improvement in anterior chamber inflammation was accompanied by a decrease in the brightness of the iris vessels, up to the point in which at 14 days OCTA is no longer able to detect them. Bottom row in Fig. 7 reports the difference in pixels brightness in the various portion of the iris at the three time points.

#### Quantitative analysis using automated algorithm

OCTA images of the iris (Fig. 3a) are processed using a combination of 3D Gaussian filters, spectral bandpass filters, intensity thresholding, and 3D morphological “opening” filters (Fig. 10b) to remove non-specific, non-contiguous voxels and segment bright, connected voxels representing vessel segments (Fig. 10c). Vascular volumes are subsequently calculated by summing voxels in the resulting vessel masks. A mean 3 × 3 vascular iris volume in patients with 4+ anterior chamber cells was 0.28 mm^3^, in 3+ cells 0.19, in 2+ cells 0.11, in 1+ cells 0.08 (Video 1).

## Discussion

Since its introduction in the market, OCT has deeply changed the clinical practice of most of the subspecialty in ophthalmology.

Along the years, this has evolved from a pure morphologic diagnosis test to a functional non-invasive technology that is gradually gaining a progressive consideration among the traditional diagnostic methods.

The pioneering approaches to a series of inflammatory eye conditions, such as pterygium, NVV and, more interestingly, anterior uveitis, has exerted a strong impact to both the therapeutic approach and the pathophysiology understanding of such diseases.

Among the different diseases, anterior uveitis is the one presenting trickier aspects, due to its multifactorial component and the complexity of the involved anatomical structures.

The signs of intraocular inflammation in vivo include the infiltration of white blood cells into the AC and posterior vitreous cavity, iris vascular dilation, dilation and inflammation of the walls of the retinal vessels, and an increase in the protein concentration in the intraocular fluids. The relationship between posterior uveitis and inflammatory changes in the retinal vessels are well established and can be assessed using fluorescein angiography [28, 29].

As per pathophysiology, the iris and ciliary body are the primary sites of inflammation in anterior uveitis [1], but ciliary body is not visible due to its anatomical location, the iris can be imaged with both indocyanine angiography[9, 10] and, now, OCTA. The surface of the iris shows a varying number of depressions and ridges, created by vessels in the stroma, generally running radially from the pupil toward the base of the iris and occasionally extending obliquely or circumferentially. The blood vessels of the iris run radially in an unusually sinuous or coiled manner. They enter the iris root and pass through the ciliary zone in several layers. At the junction between the ciliary and the papillary zones, they anastomose to form the minor circle of the iris, which consists of both arteries and veins. A thick adventitia formed by a collar of collagen fibers and the absence of fenestrated capillaries account for the low permeability of iris vessels. Most of these vessels have a continuous endothelium whose cells are joined by complex zonula occludens and gap junctions. For this reason, past fluorescein angiography studies of the iris in uveitis have not shown leakage of the dye from the iris vessels [12].

In order to better monitor patients clinically and to determine their response to treatment in clinical trials, they have been working to develop objective measures of ocular inflammation using multi-modal imaging [20–22]. Pichi et al. [26] have shown that the iris vascular dilation and blood flow increase can be identified and quantified on OCTA of the iris in patients with AAU. This pilot study suggests that iris vascular caliber might be a good candidate for quantification given the increase seen in the presence of inflammation and the improvement in response to treatment. Those findings are in accordance with a previous paper by Choi et al. [25], who employed in vivo OCT microangiography in rodent iris. With this method, the authors were able to non-invasively describe iris vascular anatomy in rats and to register a caliper increase in those same vessels when uveitis was induced in the rodents. Even if their results of vascular dilation in the iris following inflammation are consistent with ours, Choi et al. [25] manually measured and compared the vessels caliper at different time points, whereas in the current study an objective quantification was employed.

We identified a few challenges that will need to be addressed in future OCTA angiography studies of the anterior segment. First, the custom-made system does not have sequential image registration capacity, which led to a difficulty in precisely locating the same region of interest for day 0, day 7, and day 14 images. Second, for anterior OCT imaging, ocular tissue refraction of the OCT beam can distort the physical geometry of the anterior segment in an OCT image. The reliability of these quantitative parameters can be improved with correction of the optical distortion considering the refractive indices of ocular tissue layers. We do not feel that this changes with treatment but would need further study to confirm that refraction does not change with improvement in the degree of ocular inflammation or with treatment with steroids. If it can be shown that it does not change, this effect can be ignored for each individual patient and the degree of change in can be quantified; however, it would need to be considered to compare measurements between patients or between the two eyes of the same patient. Third, the iris vasculature caliber and tortuosity could be affected by pupillary dilation. Although different studies try to use the same lighting conditions (ambient illumination) for all studies, a variation in pupil size is unavoidable. The pupil size needs to be constant through all imaging sessions to minimize the effect due to environmental factors that may affect iris vasculature calibers, so that the quantitative measurements of iris vessel diameter in inflammation and treatment can be reproducible. Addressing these challenges will result in improving this technique for future large-scale and long-term studies. The reduction that OCTA was able to detect in vessels diameter, density, and overall appearance, may certainly be due to the control of inflammation. However, topical steroids such as prednisolone acetate 1% have specifically profound antiangiogenic, and vasoconstrictive effects in addition to relieving inflammatory responses [30]. Therefore, an important role in the overall decrease in iris vessel diameters may be played by the vascular effects of to the steroid applied to the inflamed eye.

In summary, the current literature shows that OCT angiography is more than a promising method in identifying vasculature anomalies in patients with active anterior segment inflammatory and para-inflammatory conditions. Of course, these results have to be interpreted with limitations of a technique which is daily evolving. Future studies including eye tracking and automated quantification of the structural and vascular changes in the anterior segment of eye are the natural perspective of such diagnostic method. This may prove to be not only a useful tool but also this might represent a safe and more precise technique for the investigation of the pathophysiology of ocular inflammation and the preclinical testing of new therapeutic treatment strategies for uveitis.

## Abbreviations

OCTA: Optical coherence tomography angiography
OCT: Optical coherence tomography
AAU: Acute anterior uveitis
NVI: Neovessel of the iris
ICGA: Indocyanine green angiography
FA: Fluorescein angiography
RVO: Retinal vein occlusion
DR: Diabetic retinopathy
